# Prediction of Trace Metal Distribution in a Tailings Impoundment Using an Integrated Geophysical and Geochemical Approach (Raibl Mine, Pb-Zn Alpine District, Northern Italy)

**DOI:** 10.3390/ijerph18031157

**Published:** 2021-01-28

**Authors:** Nicolò Barago, Stefano Covelli, Mara Mauri, Sara Oberti di Valnera, Emanuele Forte

**Affiliations:** 1 Department of Mathematics & Geoscience, University of Trieste, Via Weiss 2, 34128 Trieste, Italy; covelli@units.it (S.C.); eforte@units.it (E.F.); 2 Servizio Disciplina Gestione Rifiuti e siti Inquinati, Regione Friuli Venezia Giulia, Via Carducci 6, 34122 Trieste, Italy; mara.mauri@regione.fvg.it; 3 Servizio Geologico, Regione Friuli Venezia Giulia, Via Sant’Anastasio 3, 34132 Trieste, Italy; sara.oberti@regione.fvg.it

**Keywords:** electrical resistivity tomography (ERT), tailings impoundment, trace metals, secondary mining, remediation, ground penetrating radar (GPR), geochemical analyses, environmental contamination

## Abstract

When mines are decommissioned, tailings piles can act as sources of contamination for decades or even centuries. Tailings, which usually contain high concentrations of metals and trace elements, can be reprocessed for a secondary recovery of valuable elements with an innovative approach to a circular economy. This study offers new results for tailings ponds characterisation and chemical content prediction based on an integrated geophysical-geochemical approach. The study of the Raibl Pb-Zn tailings impoundment was done using bulk chemical analysis on borehole samples, Electrical Resistivity Tomography surveys, and Ground Penetrating Radar measurements. We found valuable and statistically significant correlations between the electrical resistivity of the mining impoundments and the metal distribution, thus providing a practical opportunity to characterise large volumes of metal-bearing tailings. In particular, these results can be useful to aid in the development of environmental monitoring programs for remediation purposes or to implement economic secondary recovery plans.

## 1. Introduction

Industrial extraction and processing of mineral resources is one of the major anthropogenic global sources of potentially toxic trace elements in the environment. In 1996 alone, the volume of global mine waste production was estimated to exceed five gigatons [1,2]. Due to the exponentially growing global demand for mineral resources (especially for Rare Earth Elements (REE) and precious metals) mining companies currently exploit even low-grade ore bodies [3], where up to 99% of the material is discarded. This requires managing high volumes of potentially toxic mine waste, and fast but reasonably accurate methods for the characterisation of large areas are essential.

The common types of solid by-product of mining and beneficiation processes of the ores usually consist of huge amounts of waste rocks and tailings. Waste rocks include non-mineralised and low-grade mineralised rock removed during extraction, usually ranging in size from gravel to sand. On the other hand, tailings are the waste portion of mined material that is separated from the target mineral during the beneficiation processes (e.g., milling and flotation), and usually consists of fine materials that still contain high concentrations of desired material but below the cut-off grade. If market prices change, there may be increased motive for future reprocessing. For example, the Ag-rich tailings processed in the early years at Broken Hill Pb-Zn-Ag mine (Australia), have recently been reprocessed due to the development in new flotation technologies [4].

Generally, wet tailings are slurried and stored in tailings impoundments. As an example of the volumes involved, in one of the world’s largest volcanogenic massive sulfide deposits at Kidd Creek, Ontario, Canada, more than 130 Mt of tailings will be stored in the pond before the expected closure of the mine scheduled for 2023 [5,6]. Possible contamination due to failure of the tailing’s impoundments can and does happen, and is of global concern. From 1970 to 2011, more than 70 major impoundment failures occurred around the world [6,7] causing enormous releases of waste rock, tailings, and waste/acid waters during mining spills. In these events, enormous volumes of toxic materials can be released into the water catchment area, which happened in Val di Stava, Italy, 1985 (180,000–200,000 m^3^ [7,8]); in Aznalcóllar, Spain, 1998 (4–5 Mm^3^ [9]), and at the Mount Polley mine, Canada, 2014 (25 Mm^3^ [10]). Even several years after the closure of the mines the tailings impoundments must be monitored and protected against weathering (collapse, erosion, leaching, etc.) to safeguard environmental health at mines sites and downgradient areas.

Nevertheless, with the decommissioning of the mines, waste rock piles and tailings impoundments can act as sources of contamination at the site for decades or even centuries. Dispersion of contaminants is mainly caused due to the oxidation processes of sulfide minerals, when exposed to atmospheric conditions [11] with the subsequent release and dispersion of potentially toxic trace elements into riverine systems and downstream zones.

As a consequence, there is great interest in developing new and fast methodologies to study the internal structures, physical and chemical properties of tailings impoundments, both for environmental or economic purposes. Generally, the most accurate information on subsurface materials is obtained by borehole drilling. However, costs associated with high-resolution borehole sampling and chemical analysis are expensive, time-consuming and limited in horizontal resolution due to the obvious limitations in the number and spatial density of the drilled boreholes. The geophysical methods include different non-destructive techniques which can extend borehole punctual data, achieving 2D or 3D distribution maps by which wide areas can be characterised.

Various authors employed geophysical electrical methods [12] on tailings impoundments, also integrated with geochemical characterisations from borehole samples [13,14,15]. The integrated approach usually allows one to delineate the tailings volumes, some hidden structures, as well as bedrock or basement geometries of the impoundments. With a 3D electrical resistivity tomography (ERT) survey, Martín-Crespo et al. were able to identify and calculate the volume of the tailings currently stored in the Brunita mine pond, Spain [16], but few articles, as is the case of the phosphorogypsum pond of Vásconez-Maza et al. [17] predict the spatial distribution of metals through non-linear regression modelling combining metal concentrations and quantitative geo-physical parameters.

Ground Penetrating Radar (GPR) techniques have been used in a plethora of applications at different scales (e.g., Daniels, 2004 [18]). As for the topic of this paper, there are several examples of applications strictly related to mineral resource evaluations. A comprehensive review about the GPR applications for mining geophysics can be found in Francke, 2010 [19] and there is an interesting case study on bauxite deposits in Erten et al., 2013 [20]. More specifically, GPR allows for the imaging of the shallow subsurface of tailings dams (Porsani et al., 2019 [21]) providing qualitative information regarding their internal structures and “anomalies”.

The aim of this work is therefore to characterise high volumes of high-metal content mine tailings integrating geophysical and geochemical methods, and to use regression models to estimate the concentrations of buried tailings, cross-validating the results and in turn implementing an approach which reduces the overall time and cost of characterisation, providing necessary information for the remediation of contaminated sites, or for secondary economic recovery of valuable elements.

## 2. Materials and Methods

### 2.1. Study Area

**Figure 1:**
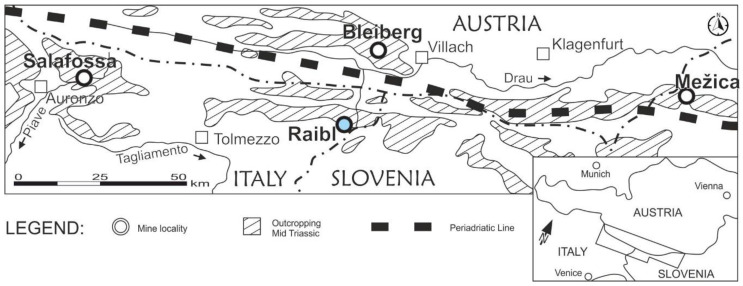
Location of the four main Pb-Zn carbonate-hosted ore deposits in the SE Alps domain, redrawn from Brigo et al. [23].

The Eastern Alps Pb-Zn carbonate-hosted province is a millenary mining area where extraction has been carried out since ancient times [22]. The Pb-Zn Raibl mine is located in Cave del Predil (Friuli Venezia Giulia, Italy-lat. 46.44150; lon. 13.56904), close to the borders with Slovenia and Austria. The Raibl mine district encompasses other carbonate-hosted Pb-Zn ore deposits along the Periadriatic tectonic Line, such as Bleiberg (Austria), Mežica (Slovenia), and Salafossa (Italy) [23] (Figure 1).

The strata-bound Mid-Upper Triassic Pb-Zn Raibl ore deposit is nestled in a 1000 m thick Ladinian carbonate massif (“Sciliar/Schlern Formation”). The Sciliar/Schlern Fm. is limited by the overlying basinal Carnian units (Raibl group) and at the base by Early Ladinian tuffs and ignimbrites of the “Rio Freddo Volcanics” [23].

The mine extends for a maximum depth of 520 m underground, with a total length of more than 120 km of underground galleries. Extraction and processing of the Pb-Zn ores in the eastern Alps has been documented since 1006 A.D. but were likely carried out since the Roman period. The mine was initially mainly an open pit and then expanded to underground mining, with the maximum production reached in the 20th century. During its final active years almost 350,000 tons/year of raw material of ore with an average ore grade of 5% Zn and 1% Pb [23] was mined in Raibl. At present, the deposits are of sub-economic interest and Raibl mine was decommissioned in 1991, like most of the Italian and Western European metal mines which closed before the end of the 20th century.

**Figure 2:**
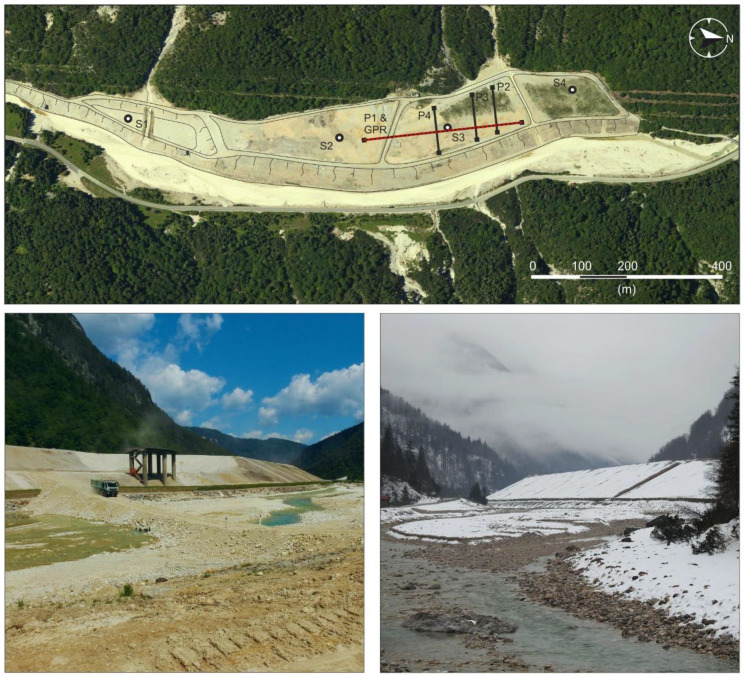
Satellite image showing the extension of the Raibl mine impoundment (**above**). Labels S1-S4 mark the location of the boreholes used in this study, while the solid black and dotted red lines with labels P1–P4 depict the ERT and GPR survey locations respectively. The two pictures below show the impoundment as it appears from the Rio del Lago stream bed, from the south (**left**) and from the north (**right**).

Based on the available documentation, after grinding, milling, and flotation separation, the tailings from the washery were directly discharged into the Rio del Lago stream waters until 1952. Only in the last nearly twenty years before the mine closed (1972–1991) tailings were slurried and stored in an impoundment (Figure 2) built at the base of Monte Re onto the Rio del Lago stream bed, which is composed of alluvial gravel with sands and boulders mainly made up of limestones and dolostones. The whole areal extension of the impoundments is approximately 130,000 m^2^, with a height ranging between 22 m and 15 m and a volume of waste rocks and tailings of almost 2 Mm^3^ (i.e., ~4 Mt).

The mill tailings are confined by external permeable dams built using waste rocks. Tailings grain size ranges from fine sand to coarse silt, with a smaller component of non-plastic clay (0–22% [24]). As the result of milling and concentration of the sulfidic ore deposit, the tailings reflect the mineral composition of the ore deposit where the mineral assemblage consists of (1) primary minerals: sphalerite, galena, iron sulfides (mainly pyrite, marcasite, and melnikovite), (2) secondary minerals: smithsonite, cerussite, hydrozincite, iron oxyhydroxides and (3) rare accessory minerals such as jordanite and gratonite [25]. Dolomite, calcite, and baryte prevail among gangue minerals.

It is interesting to note that the Julian pre-Alps sector is one of the rainiest areas in Italy and in Europe, with maximum precipitation up to 3100 mm/year. In the village of Cave del Predil, where the mine site is located, annual average precipitation is approximately 2200 mm/year, with summer and winter as the driest seasons, and autumn and spring as the rainiest [26,27].

### 2.2. Geophysical Surveys

GPR is a geophysical technique that uses the propagation of electromagnetic waves produced by antennas to image underground electromagnetic property contrasts, reconstructing their geometries. Data was acquired using a ProEx GPR system equipped with 250 MHz ground-coupled bistatic Malå Geoscience shielded antennas, with a constant transmitter-receiver offset equal to 31 cm. A constant trace interval equal to 10 cm was assured by an electro-mechanical odometer triggering the GPR system. The maximum depth depends on the penetration capacity of EM waves, which is inversely related to EM wave frequencies, to signal-to-noise ratio and, most importantly, to the EM physical properties of the subsurface related to the intrinsic attenuation of the media. In particular, the higher the electrical conductivity (which is inverse of the resistivity), the higher the attenuation of the EM wave will be and thus the lower the penetration depth will be (for a fixed antenna frequency range). The applied GPR processing flow includes: DC removal, zero time correction (drift removal), spectral analysis and filtering, geometrical spreading correction, exponential amplitude correction, depth conversion. In order to achieve depth conversion, it is essential to estimate the EM velocities of the subsurface materials. This was done by fitting diffraction hyperbolas related to scattering phenomena. Due to the limited number of pure diffraction events on the GPR dataset and their non-uniform spatial distribution, we estimate a uniform velocity field equal to 9 cm/ns. Even if this is an oversimplification, it is accurate enough since the lateral EM velocity variations are expected to be limited due to the nature of deposits. Further details on GPR data processing can be found in Jol, 2009 [28].

The real resistivity data are obtained from the inversion (finite element method, [29,30]) of the apparent resistivity dataset. The ERT survey was performed with a Syscal Pro georesistivimeter (IRIS international) and data were processed with Iris Instruments PROSYS II and inverted in RES2DINV (Geotomo software). The Wenner-Schlumberger array was identified as the best option because of its sensitivity to both vertical and horizontal variations, being a good compromise between the Wenner and the Dipole-Dipole arrays [30]. Data are generally characterised by high quality measurements and a minimum number of outliers had been eliminated. Electrode-soil coupling was optimised due to the presence of a cover made of 50 cm of fine material, deployed during the remediation operations on the tailings ponds to minimise runoff water infiltration rates and the risk of aerial dispersion of tailings, mainly in the direction of the village of Cave del Predil. The real resistivity data are obtained from the inversion [29,30] of the apparent resistivity dataset. In the present case, For ERT data analysis and inversion we used Prosys II software (Iris instruments) integrating for the inversion the ERTLab suite (Multi-Phase Technology and Geostudi Astier) and Res2Dinv (Geotomo software), using tetrahedral finite element modelling. After a data quality check and selection, considering both reciprocal measurements, thresholds on the minimum current and voltage, as well as on the maximum percentage RMS error was limited to 2%, we inverted the ERT dataset by using a smoothness constrained least-squared algorithm. ERT inversion was limited to a maximum data depth of 25 m, allowing for the exploration of the entire section of the impoundments.

Four ERT profiles and a GPR survey were done in September 2018 on a portion of the Raibl tailings impoundment (Figure 2). Both ERT and GPR data was collected along profile P1. Along profiles P2, P3, and P4 only an ERT survey was done. The coordinates of each survey were recorded using a DGPS measuring the position of each individual electrode. The P1 survey (Figure 2) consists of a 332 m long Wenner-Schlumberger array composed of three concatenated profiles with partial overlapping between each other. Each profile has 72 stainless electrodes spaced 2 m apart. The overall high quality of the data is evidenced by the standard deviation of the measurements: for example, for the entire P1 profile (3663 measurements) there were only 6 values (removed before the inversion) exceeding a standard deviation of 3%. The RMS error after the inversion of the three concatenated profiles of P1 is 8.2% after 4 iterations (Res2Dinv software), however, the RMS of three singularly inverted profiles is always lower than 5%. P2, P3 and P4 surveys cross perpendicularly P1 (Figure 2) and each profile consists of a 94 m long Wenner-Schlumberger array composed of 48 stainless electrodes 2 m spaced apart. The maximum investigation depth is equal to approximately 20 m. The RMS error after 4 iterations is 3.9%, 2.0% and 3.1%, respectively for P2, P3 and P4.

### 2.3. Sample Collection and Geochemical Analysis

Four boreholes (S1, S2, S3, S4) were drilled in the impoundments in 2007. The maximum depth of the boreholes was chosen in order to reach at least 3.5 m below the tailings beds. Profile P1 was designed to cross the location of borehole S3 (Figure 2). No fluids were used during drilling so as to avoid contaminating the samples. To obtain representative vertical information, the boreholes were subsampled in 1 m long sections and then homogenised and sieved at 2 mm. Sample digestion was conducted in *aqua regia* (1:3, HNO_3_: HCl) and H_2_O_2_ following the EPA 3050B method with an Anton Paar Multiwave 3000 whereas element concentrations were measured by Inductively Coupled Plasma-Mass Spectrometer (ICP-MS) according to UNI EN ISO 17294-2:2005 procedure at the Regional Environmental Protection Agency (ARPA FVG) laboratories. The accuracy and precision of our results were checked by analysing the standard reference material (NIST SRM 2709—San Joaquin Soil). Acceptable recoveries were obtained ranging between 82% and 124%. The precision of the analysis expressed as RSD% was < 3%.

## 3. Results

### 3.1. GPR and ERT Results

**Figure 3:**
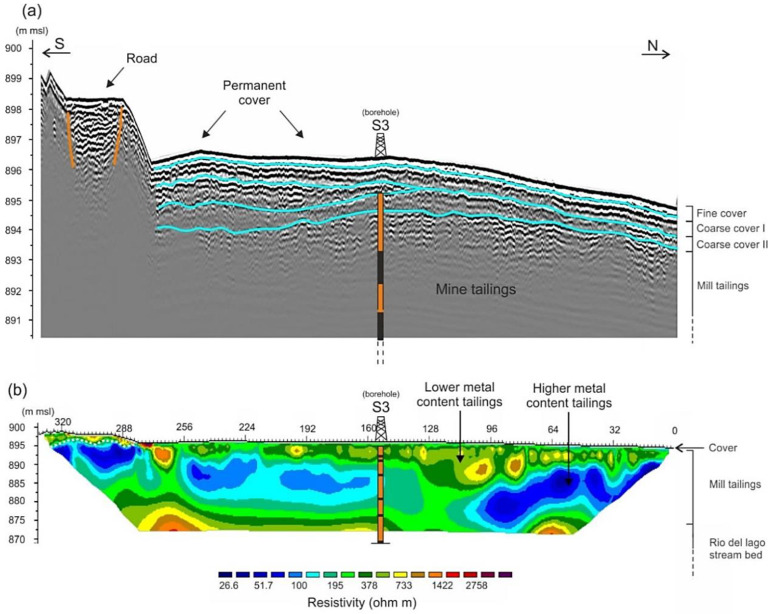
(**a**) GPR profile P1; (**b**) ERT profile P1. The samples analysed from the s3 borehole are reported in Figure 5.

GPR data allow for the imaging of four main continuous sub-parallel horizons with local bending and lateral closures (Figure 3a). The maximum depth reached by EM waves was equal to approximately 3 m. From the surface, the first layer of 50–70 cm is observable, although it partially interferes with the ground wave. This layer is interpreted as the fine cover deployed on the pond surface during the permanent sealing operations designed to reduce runoff water infiltration and aerial dispersion. Below it, two other layers of minor amplitude represent two other coatings deployed at different times, most probably constituted by coarse material and waste rock. Below, a homogeneous undefined layer of mill tailings can be recognised, but the EM waves can penetrate this layer for no more than a couple of meters as the nature of the tailings leads to the rapid attenuation of the signals [31]. The above-described results reveal a regularly stratified sequence, at least in the shallower portion of the deposit and is related to the mining activity in which a sequence of levels accumulated over time filling in the tailings basin at varied intervals.

**Figure 4:**
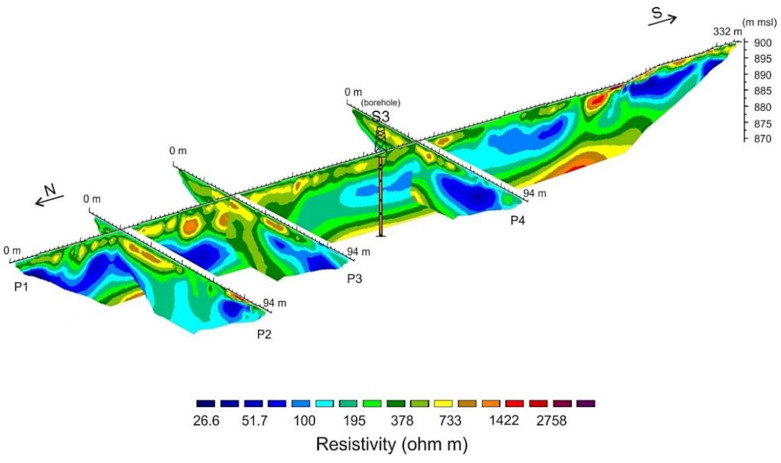
3D view of inverted resistivity data on the Raibl tailings impoundment.

**Figure 5:**
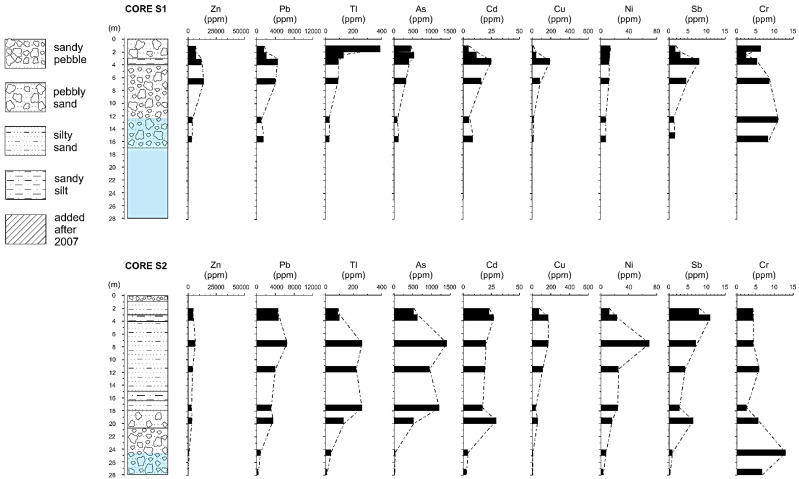

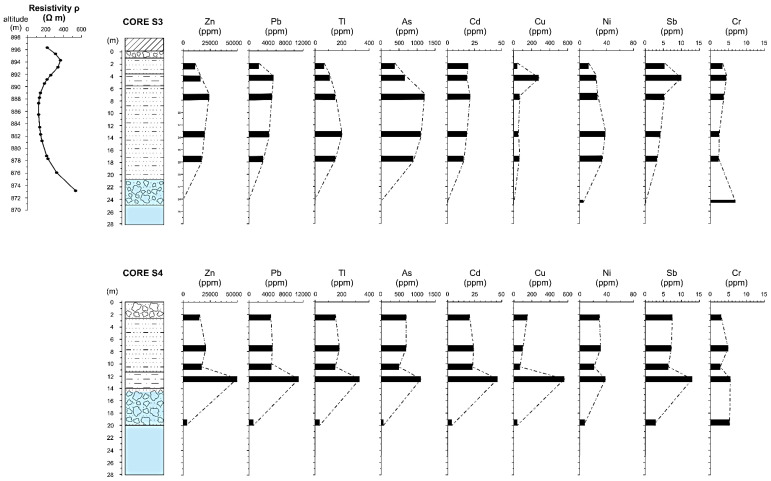
Stratigraphy and vertical profiles of trace element concentrations in the S1, S2, S3, S4 boreholes drilled on the Raibl tailings impoundment. Water table levels (in light blue) refer to values for June–July 2007.

Profile P1 (Figure 3b), acquired along the same path of the GPR profile, represents the inverted resistivity section of the impoundment. Borehole S3 is also seen in Figure 3b which assisted in the interpretation/validation of the geophysical data. The 3D view including ERT profiles P1, P2, P3, and P4 of the Raibl impoundment is shown in Figure 4. It is interesting to note that the resistivities at the crossing points are in good agreement between the different profiles and that the lateral variations are less relevant than the vertical ones. In contrast to the GPR technique, the ERT survey reached maximum depths of approximately 25 m, thus preventing any integrated analysis or joint inversion. Most of the resistivity values indicate medium-low resistivity materials (<400 Ohm m) which correspond to the fine mill tailings produced after grinding, milling, and flotation of the ore. The mill tailings layer can be divided into two discontinuous sub-layers. The most superficial layer (100 < Ohm m < 400) (Figure 5), and the deeper layer (30 < Ohm m < 100) characterised by very low values of electrical resistivity. Below the tailings beds, at 20-25 m, an irregular layer exhibits values of electrical resistivity > 1000 Ohm m. This is interpreted as the substrate of the tailings, composed of alluvial sediments from the Rio del Lago stream, alluvial fans from the Monte Re possibly with thin subsoil layers.

### 3.2. Geochemical Characteristics of the Tailings

Geochemical results and stratigraphic sequences of the four boreholes (S1, S2, S3, S4) are shown in Figure 5. The average composition of Pb and Zn in tailings published in this study are similar to the tailings compositions reported by the “SIM” mining company in 1988 [32] (Table 1).

Geochemical vertical profiles showed that tailings deposits may host valuable amounts of metal(oid)s available for secondary mining and their presence can represent a notable potential source of contamination. The tailings, being the result of grinding and flotation of the ore, reflect the chemical composition of the ore deposit [33]. They are strongly enriched in Zn and Pb as well as in trace elements such as As, Tl and Cu. Other metals such as Fe, Ge and Ga are reasonably present in detectable or significant concentrations [34], but they were not determined in our analyses. Geochemical profiles showed maximum amounts of metals and trace elements in the sandy and silty tailings beds, reaching concentrations up to 5% Zn, 1.1% Pb, 1400 ppm of As, 560 ppm of Cu, 330 ppm of Tl and 46 ppm of Cd, respectively. On the contrary, except for Cr, the lowest concentrations always correspond to the tailings bed substrate. The vertical variability of Zn, Pb, Tl, As, Cd concentrations is similar in the four boreholes, except for Cr, since its occurrence is independent of the ore-component.

The lateral and vertical heterogeneity of metal distribution in the Raibl impoundment is noticeable. From 3D ERT data and chemical profiles, the higher-grade areas of tailings appear located approximately in the central area of the impoundments, whereas the most surficial and deepest portions of the tailings beds seem depleted of metals. The oxidation of sulfides from rainfall and runoff waters in the uppermost part [35] could be a possible explanation whereas the water table oscillation could be responsible for metals scavenging in the lowest part of the impoundment. However, the heterogeneity can also be explained by differences in the methods used to fill in the tailings ponds over time, the composition of the ore source, particle size distribution [35], upgrading of beneficiation techniques, and/or differential metal depletion from weathering/oxidation of sulfide minerals.

### 3.3. Relationships between the Geochemistry of Tailings and Resistivity

**Figure 6:**
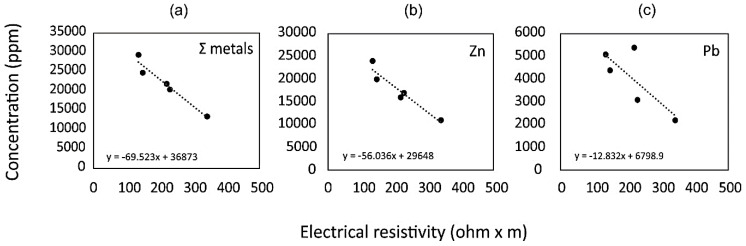
Scatterplots between metal content in the samples from borehole S3 vs. ERT inverted resistivity values. (**a**) Sum (Σ) of metals include Zn, Pb, Tl, Cd, Cu, Ni, Sb and Cr whereas only Zn and Pb concentrations are reported in (**b**) and (**c**), respectively.

In this study, despite the relatively small amount of available geochemical data in terms of areal distribution, empirical correlations between inverted resistivity and the chemical composition have been found. Since the deepest section of borehole S3 is below the maximum ERT depth, laboratory data are framed in the ERT range. In Table 2 and Figure 6, respectively, the Pearson coefficients and scatterplots between the inverted resistivity of the tailings and the related concentrations of the main metal(oid)s in borehole S3 are shown. It is apparent that the sum of metal (Zn + Cr + Tl + Cd + Cu + Ni + Sb + Cr) concentrations is significantly correlated with the inverted resistivity (r = −0.975, *p* < 0.01) confirming that metal content is the main factor which increases the conductivity in tailings. Among the elements, Zn, which is the most abundant, shows the best correlation with resistivity (r = −0.958, *p* = 0.01). The correlation coefficients of the other elements are variable, but always lower than Zn. Unfortunately, Fe was not analysed since the aim of the geochemical characterisation of the boreholes was to identify the concentrations and vertical distribution of potentially toxic elements only.

### 3.4. Estimation of Metal(oid) Content

**Figure 7:**
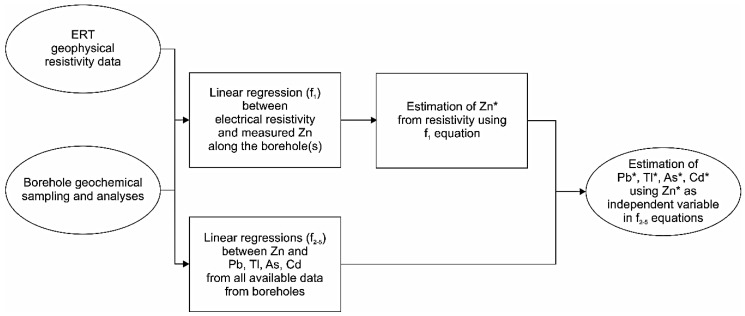
Flux diagram of the procedure for the estimation of metal content. For f_n_ meaning refer to the equations reported in Table 3 and ERT as “Electrical Resistivity Tomography” technique. The notation “*” indicates the estimated metal(oid) concentrations.

**Figure 8:**
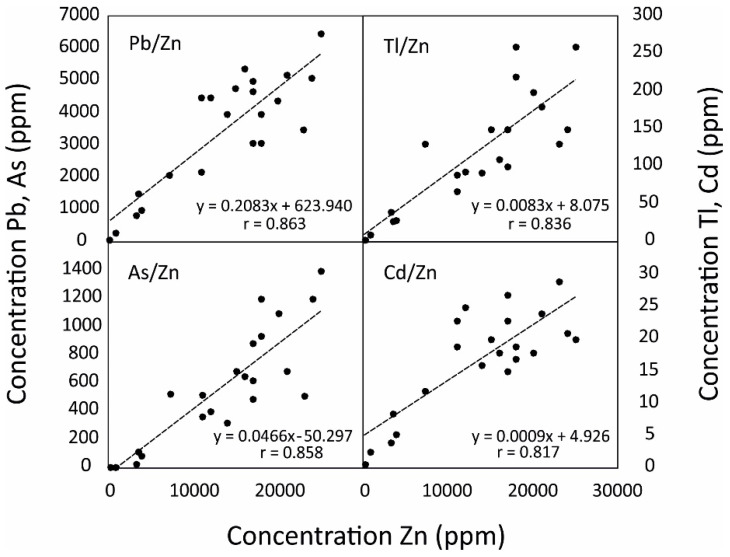
Scatterplots of the borehole samples with the related regression lines between Zn and Pb, Tl, As and Cd concentrations.

The modelling procedure includes two processing steps. The first step allows for the estimation of the Zn content (Zn*) in tailings based on the inverted resistivity survey data, and the second step estimates the other trace element concentrations starting from the estimated Zn* concentration, as shown in the diagram in Figure 7. Zn was chosen because it shows the best linear correlation with both resistivity and metal(oid)s such Pb, Tl, As and Cd (*r* = 0.863, 0.836, 0.858, and 0.817, respectively; *p* < 0.001; Figure 8). Moreover, the correlations among Zn and the cited elements are consistent with the Alpine Pb-Zn carbonate-hosted deposits found in the literature, since it is known that sphalerite minerals (ZnS) usually host a notable amount of (potentially toxic) trace elements especially Tl and Cd [25,34,36].

The empirical linear function between resistivity and the observed Zn concentration, along the borehole S3 (Figure 6b), has used to obtain the estimated Zn content (Zn*) from ERT inverted resistivity data (see Table 3; equation f_1_). Then, in second step, after the elimination of the outliers (N_outliers_ = 3), the site-dependent empirical linear function between Zn and Pb, Tl, As, Cd concentrations, calculated from all the samples available in the boreholes (S1, S2, S3, S4), were obtained (Figure 8). These linear function parameters were used to predict metal(oid) concentrations (Pb*, Tl*, As* and Cd*) from Zn* estimated content (Table 3, equations f_2–5_).

These steps allow for the calculation of chemical concentrations purely from resistivity data after the previous correlation/validation.

**Figure 9:**
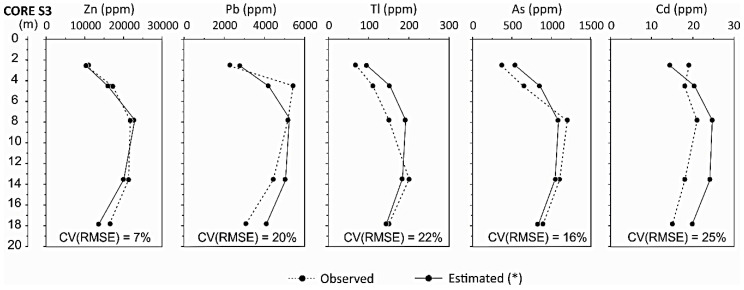
Estimated concentrations of the main metals. Zn* concentrations result from the inverted resistivity values of the ERT geophysical survey, Pb*, Tl*, As*, and Cd* concentrations result from the calculated Zn* concentrations.

The coefficient of variance of the root mean square error (CV(RMSE)) indicate that errors of 7–25% can be obtained during the estimation of metal/trace element content (Figure 9) in the Raibl impoundment. This results in the acceptable accuracy of the estimation method proposed, providing a simple modelling approach that can be used to extend the borehole geochemical composition over wide volumes of tailings.

## 4. Discussion

Considering the extension and the volume of the impoundment area, absolute resistivity data can vary greatly from site to site, since electrical properties are strongly affected by site-dependent characteristics. For instance, Placencia-Gómez et al. [14] found values between ~ 5 and 30 Ohm m in the Haveri Au-Cu tailings impoundments, whereas Martínez-Pagán et al. [37] reported < 8 Ohm m values for Brunita Pb-Zn mine impoundment. However, our resistivity data are more coherent with Cortada et al. [31] who found values below 150 Ohm m for Pb-Ag and Cu-Fe Linares-La Carolina tailings ponds in unsaturated conditions.

In other studies, regression models have been successfully used to estimate physical or chemical properties of the subsurface based on empirical correlation from in situ ERT resistivity data. For example, Vásconez-Maza et al. [17] predicted Cr distribution from ERT in a phosphorogyspum pond near Cartagena, Spain (R^2^ = 0.68), and Alamry et al. [38] pointed out lithology-specific exponential correlation (up to R^2^ = 0.98) between soil moisture and ERT. In this study, a strong correlation was found between ERT and the sum of metal concentrations and Zn (R^2^ = 0.95 and R^2^ = 0.92, respectively) from the Raibl Pb-Zn impoundment. We believe that the main factor influencing the resistivity is the metal content, as evidenced by the strong correlation. An important factor can be the high electrical contrast between conductive fine metal-bearing tailings and non-conductive gangue materials and minerals. Therefore, the majority of the fine particles in the impoundments are exclusively the conductive milled metal-bearing tailings in contrast to mixed metal-depleted waste rock/natural sedimentary material which are usually coarser, carbonatic and more resistive.

We exclude water content as the primary influencing factor of the resistivity values because the ERT survey was performed during a dry period where the water level was below the depth of the samples used to correlate the ERT. Similar hydrological conditions were found by Vásconez-Maza et al. [17] who revealed strong correlations of Cr in the absence of the water table. In the present study, the absence of saturated sediments prevents the camouflage of metal signals, whereas saturated conditions would have resulted in scarce or unrealistic correlations between metal content and resistivity values.

A certain amount of clay usually influences resistivity values as reported by Alamry et al. [38]. The authors pointed out that the best empirical correlations with ERT data were found in sites with lower clay content and thin topsoil of the substrate. On the contrary, the presence of clay (sensus: minerals) results in a very low coefficient of determination (R^2^) between resistivity and physical properties, and the data points are scattered. In our case clays are scarce (2–20%) and result from grinding operations of the ore and the carbonatic wall rock. Hence, in this case, it is reasonable to suppose that the electric influence of these clays is very limited with respect to, for example, marine or alluvial mineral clays.

The absence of a water table (i.e., of saturated sediments) within the depth considered for correlations between geophysical and geochemical data makes such a link more robust, as previously noted. On the other hand, the presence of water in the vadose zone, which is always present even considering the climatic characteristics of the study area (see last paragraph of Section 2.1), can increase the mobility of ions within the quite porous and permeable materials (permeability k ≥ 10^−1^ cm s^−1^ [39]) of the tailings impoundment mainly through electrolytic conduction mechanisms.

The proposed two-step linear modelling has a reasonable and acceptable accuracy in estimating the metal(oid) composition from resistivity data, also due to the intrinsic characteristics of the tailings impoundment as previously stated. However, it is important to notice that the accuracy of the estimation does not depend only on the correlation coefficients among the Zn and other metal(loid) concentrations measured in the S3 borehole and in the whole dataset of the borehole samples. Indeed, the Zn/metal(oid) ratio measured in the single borehole and considering the whole sample dataset may be different due to local heterogeneity of the tailing material. Higher bias can occur when the elemental ratios of the investigated tailings deviate from the average elemental ratios of the entire composition of the tailing deposits.

## 5. Conclusions

The integration between a geophysical ERT and GPR survey and geochemical analysis on boreholes has successfully revealed the internal spatial characteristics of the Raibl mine tailings impoundments. The limited penetration depth of the GPR signal, not exceeding approximately 3 m, allowed us to image just the shallower portion of the deposit highlighting a sequence of sub-horizontal levels accumulated over time filling in the tailings basins. On the other hand, the correlation between electrical inverted resistivity and Zn content, which is by far the most abundant among the investigated elements, is statistically significant (R^2^ = 0.919; *p* = 0.010). It is hypothesised that scarce to absent clay minerals, unsaturated conditions during the survey and the high electrical contrast between gangue and ore, due to mineral composition and grain size, could be the necessary conditions for the prediction of metal content in mine tailings impoundments after the appropriate site-dependent data correlation. Based on resistivity data, empirical linear regression was used to estimate metal(oid) content with errors between 7% and 25%. The results of this study could be helpful to develop new 3D rapid methodologies to predict metal content, implementing environmental monitoring strategies or planning possible secondary recoveries of valuable elements in decommissioned tailings impoundments.

## Figures and Tables

**Table 1:** Average (± standard deviation) flotation tailing chemical composition of the Raibl mine obtained in the present study and compared with previous results (n.d. not determined).

Authors	Zn (%)	Pb (%)	Fe (%)	Tl (ppm)	As (ppm)	Cd (ppm)	Cu (ppm)
This study	1.61±1.00	0.37±0.22	n.d.	132±94	580±382	16±9	81± 122
Technical report, 1988 [32]	1.68	0.47	5.51	n.d.	n.d.	n.d.	n.d.

**Table 2:** Pearson correlation coefficients between inverted resistivity and selected elements. Σ of metals include Zn, Pb, Tl, Cd, Cu, Ni, Sb and Cr.

Σ Metals	Zn	Pb	Tl	As	Cd	Cu	Ni	Sb	Cr
−0.975	−0.958	−0.780	−0.857	−0.951	−0.221	−0.039	−0.750	−0.085	−0.064

**Table 3:** Linear functions used in the estimation of the metal(oid) contents along borehole S3. The inverted resistivity (*ρ*) is obtained from the ERT survey, whereas the notation “*” indicates the estimated metal(oid) concentrations.

Name	Linear Functions		Data
f_1_	Zn*	= −56.036 ρ + 29648	Figure 6b
f_2_	Pb*	= 0.2083 Zn* + 623.940	Figure 8
f_3_	Tl*	= 0.0083 Zn* + 8.075	Figure 8
f_4_	As*	= 0.0466 Zn* + 50.297	Figure 8
f_5_	Cd*	= 0.0009 Zn* + 4.926	Figure 8

## Data Availability

The geophysical data presented in this study are available on request from the corresponding author. Geochemical data are not available due to agreements with the regional authorities.
